# First observation of intraocular extranodal natural killer/T-cell lymphoma secondary to a retroperitoneal tumour: a case report and comparative review

**DOI:** 10.1186/s12886-022-02362-6

**Published:** 2022-03-26

**Authors:** Binyao Chen, Shizhao Yang, Wenru Su

**Affiliations:** 1 State Key Laboratory of Ophthalmology, Guangdong Provincial Key Laboratory of Ophthalmology and Visual Science, Zhongshan Ophthalmic Center, Sun Yat-Sen University, Guangzhou, 510060 China; 2 Guangdong Provincial Clinical Research Center for Ocular Diseases, Guangzhou, China

**Keywords:** Extranodal natural killer/T-cell lymphoma, Vitreoretinal lymphoma, Diffuse large B cell lymphoma, Epstein-Barr virus, Case report, Metastatic lymphoma, Intraocular lymphoma, Differential diagnosis, The optical coherence tomography

## Abstract

**Background:**

Vitreoretinal lymphomas are difficult to diagnose due to their insidious onset and inaccessible focal points. Natural killer/T-cell derived malignancies are rare as intraocular lymphomas and usually have a rapid progression and a poor prognosis. Therefore, it is essential to make a definite diagnosis, especially differentially with B-cell-derived lymphomas, which account for most cases of vitreoretinal lymphomas.

**Case presentation:**

This case report describes a 55-year-old female reporting a 10-month history of painless decline in her vision of the right eye. Optical coherence tomography of the patient revealed hyperreflective nodules and irregular humps in the retinal pigment epithelium layer. The right vitreous was aspirated for diagnostic assessment, revealing an interleukin-10 level of 39.4 pg/mL and an interleukin-10/interleukin-6 ratio of 1.05. The right vitreous humor was positive for Epstein–Barr virus DNA. Upon a systemic examination, a high metabolic nodule was found in the retroperitoneal area and proven to be positive for Epstein–Barr virus-encoded mRNA, CD2, CD3ε, TIA-1, and Ki-67. Considering the homology of the two lesions, the patient was diagnosed with metastatic vitreoretinal lymphoma secondary to retroperitoneal extranodal natural killer/T-cell derived lymphoma. The patient received systemic chemotherapy and regular intravitreal injections of methotrexate. Her visual acuity of the right eye had improved from 20/125 to 20/32 at the latest follow-up. No new lesions were found.

**Conclusions:**

A definitive diagnosis of vitreoretinal lymphoma is challenging. On some occasions in which pathological evidence is missing, the available examination results and clinical observations must be comprehensively considered. This study herein summarized pertinent pieces of literature and reports and reviewed available practicable methods to make a definitive diagnosis of intraocular extranodal natural killer/T-cell lymphoma, which was particularly distinct from the common diffuse large B-cell lymphomas.

## Background

Vitreoretinal lymphoma (VRL), a masquerade syndrome mimicking chronic uveitis or vitritis, is mostly classified as diffuse large B-cell lymphoma (DLBCL), with a few cases being derived from natural killer (NK) or cytotoxic T cells [1]. Extranodal natural killer/T-cell lymphoma, nasal type (ENKTL), an NK/T-cell derived malignant lymphoma, is characterized by scarceness and aggression [2]. The majority of ENKTL occurs in the nasopharynx, and cases of vitreoretinal ENKTL without nasal lesions are therefore extremely rare [3]. Its rarity, along with its clinical presentations and imaging manifestations similar to those of intraocular DLBCL, might result in a delayed or erroneous diagnosis. In recent years, emerging ocular examination techniques, such as optical coherence tomography (OCT), indocyanine green angiography (ICGA), and aqueous or vitreous humoral testing, have greatly promoted the diagnosis of intraocular tumours [4, 5]. This study presents a case of recurrent glucocorticoid-resistant uveitis, which was eventually diagnosed as vitreoretinal ENKTL secondary to retroperitoneal lymphoma.

## Case presentation

A 55-year-old Chinese female was admitted for a 10-month history of painless decline in her vision of the right eye. The patient was initially diagnosed with chronic uveitis and received glucocorticoid-based therapy; however, she responded poorly and was thus referred to our hospital. She reported a transient history of eye redness but no history of photophobia, floaters, flashes, or scotoma in her right eye. During the systemic review of the patient, no symptoms of oral ulcers, vitiligo, alopecia, headaches, or joint pain were revealed. The patient did not experience any eye trauma or surgery.

**Fig. 1:**
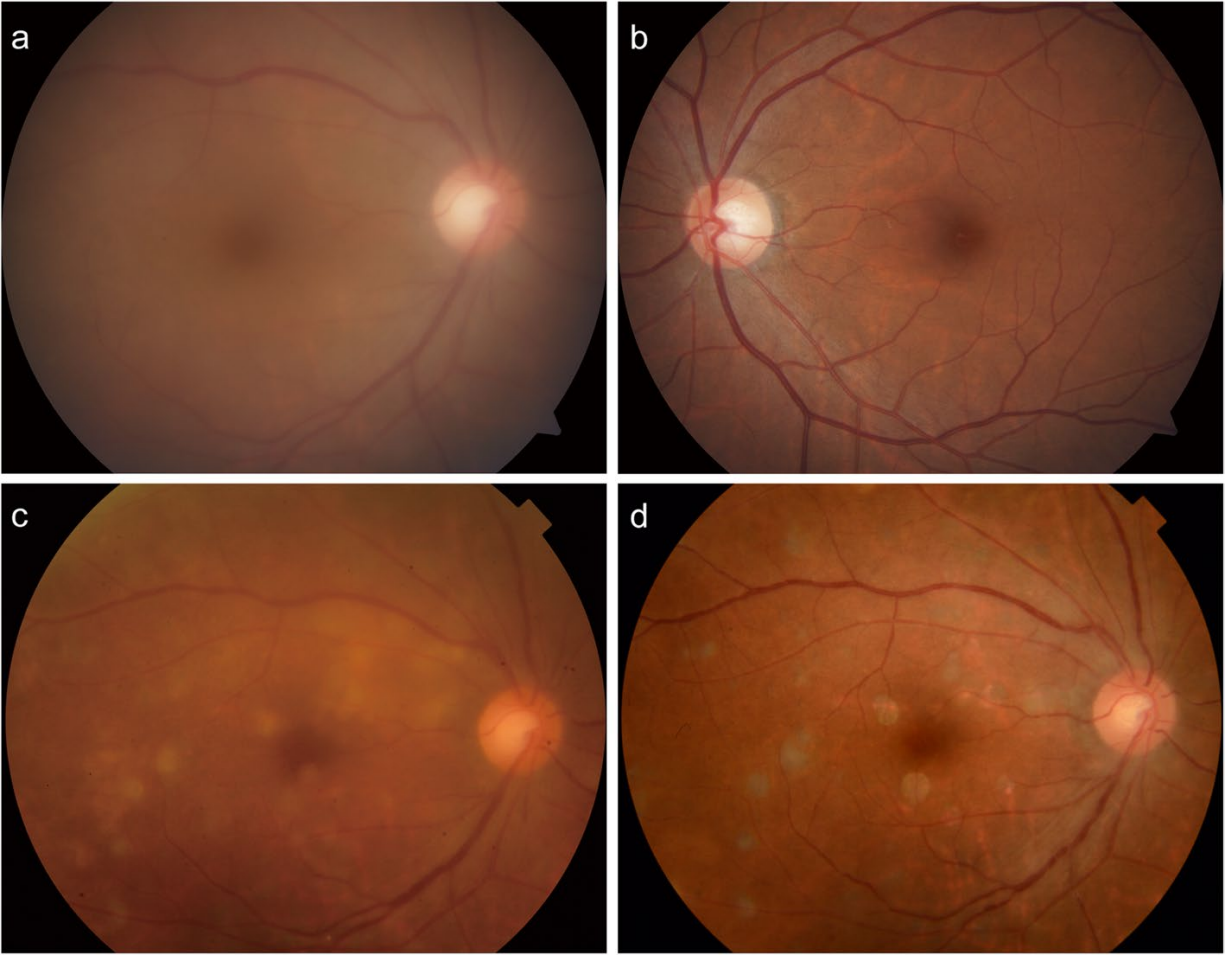
Fundoscopy images of the patient. **a** Colour fundus photograph of the right eye at the first examination displaying severe opacity of the vitreous humour. **b** Colour fundus photograph of the left eye displaying a normal appearance. **c, d** Colour fundus photograph of the right eye at the follow-ups displaying an improved vitreous opacity and multiple round or roundish yellowish-white lesions

**Fig. 2:**
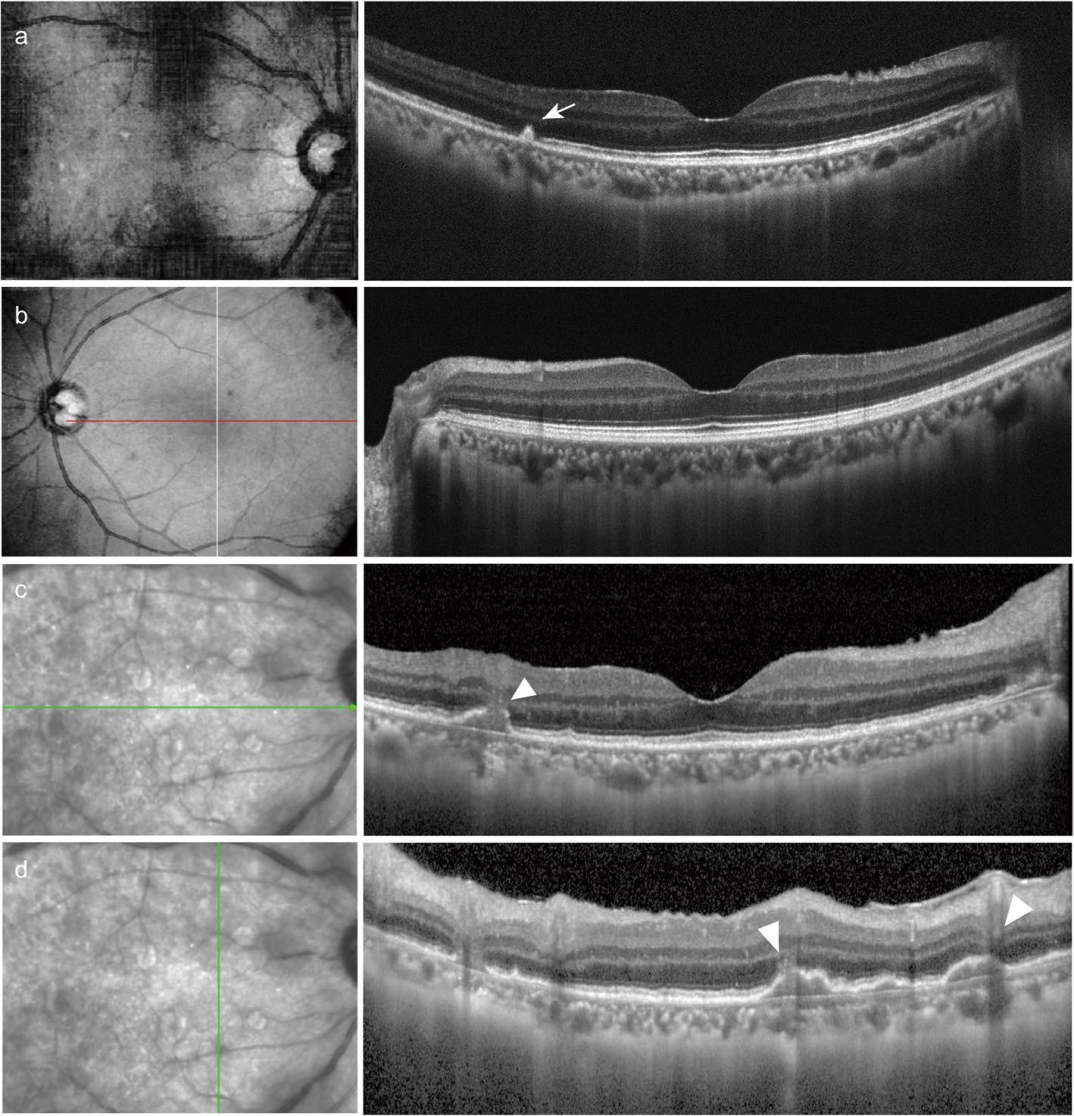
OCT manifestations of the patient. **a** OCT image of the right eye at the first examination displaying high-density reflecting spots in the RPE layer. **b** OCT image of the left eye at the first examination displaying a normal appearance. **c, d** OCT image of the right eye at the follow-ups displaying multiple irregular humps inward toward the RPE and an intact Bruch’s membrane

**Fig. 3:**
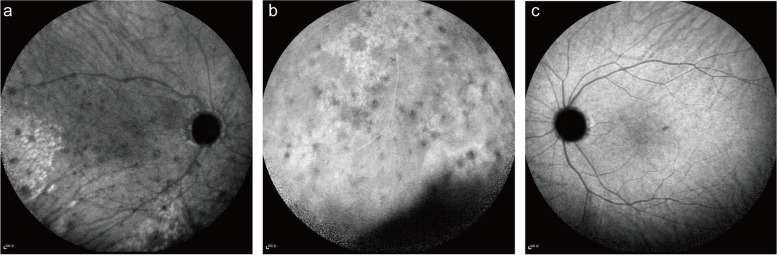
ICGA manifestations of the patient. **a** ICGA image of the right eye at the first examination displaying hypofluorescence spots and clustered hyperfluorescent points at the posterior pole. **b** ICGA image of the right eye at the first examination displaying hypofluorescence spots and clustered hyperfluorescent points at the mid-peripheral. **c** ICGA image of the left eye at the first examination displaying a normal appearance

Her ophthalmic examination revealed a visual acuity measuring 20/125 OD and 20/25 OS, and her noncontact intraocular pressure values were 13 and 15 mmHg, respectively. The results of both the extraocular appearance and muscle movement examinations were normal. The patient's pupillary light reflex was satisfactory, and her relative afferent pupillary defect was absent. Meticulous slip-lamp examination of the patient's right eye revealed diffuse mutton-fat keratic precipitates, 2 + anterior chamber (AC) cells, and 2 + AC flares. Severe vitreous opacities in the right eye obscured the dilated fundus examination (CLARUS™ 500, Carl Zeiss Meditec AG, Germany) (Fig. 1a). OCT (Cirrus HD-OCT, Carl Zeiss Meditec, Inc., United States) of the right eye showed high-density reflective spots (indicated by the arrow in Fig. 2a) in the retinal pigment epithelium (RPE) layer, while Bruch’s membrane remained intact. Spotted hypofluorescence was observed at the posterior pole and mid-peripheral retina, with clustered hyperfluorescent points at the mid-peripheral retina being observed on the ICGA OD (Fig. 3a-b). The results of fundoscopy, OCT, or ICGA of her left eye were unremarkable (Fig. 1b, 2b, and 3c).

**Fig. 4:**
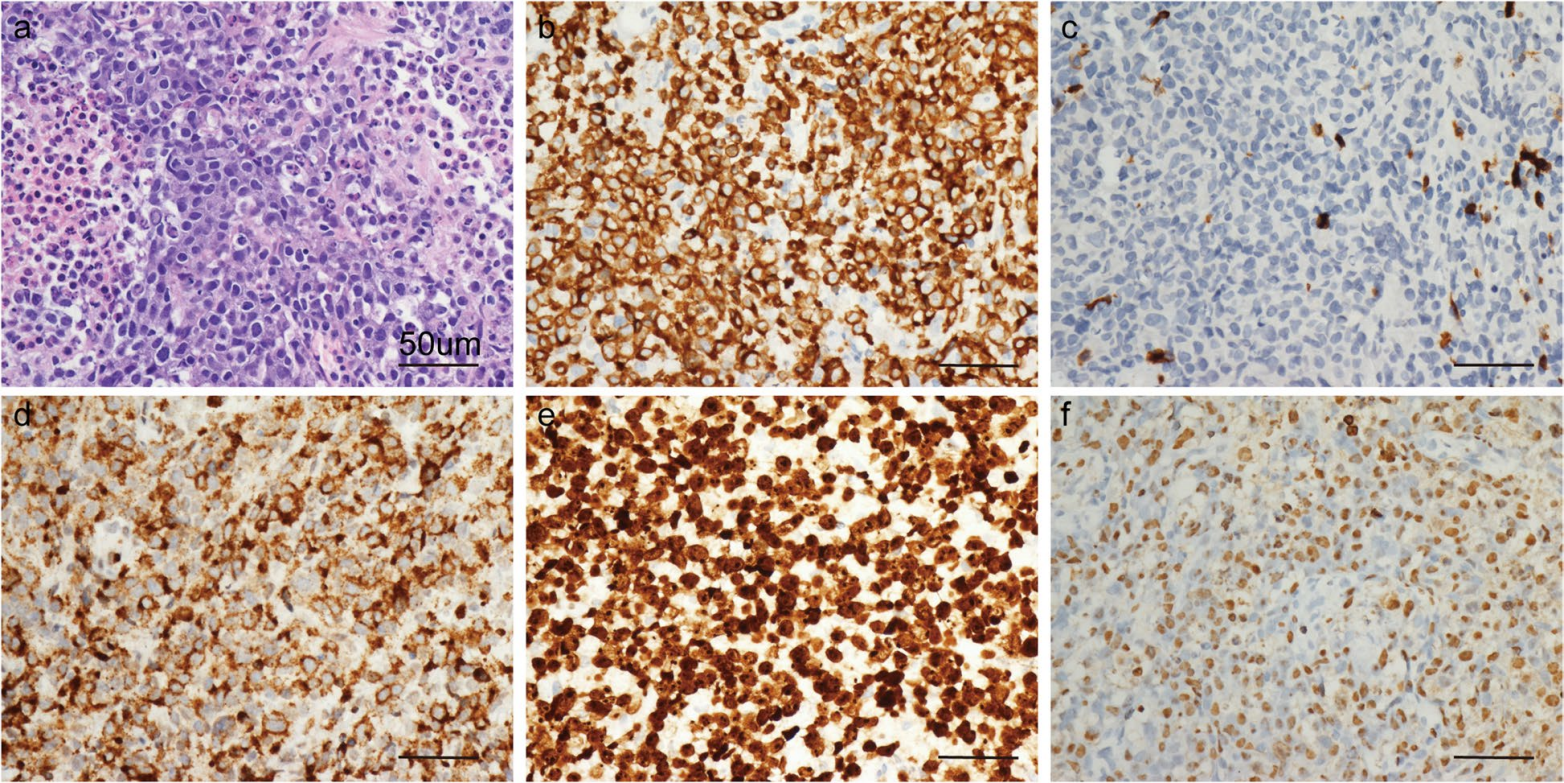
Core-needle biopsy results of the retroperitoneal mass. **a** Haematoxylin–eosin staining displaying diffuse proliferation of monomorphic lymphoid cells with irregular karyotypes, karyokinesis, and karyorrhexis. **b** Immunohistochemistry results showing CD3ε positivity. **c** Immunohistochemistry results showing CD5 negativity. **d** Immunohistochemistry results showing GrB positivity. **e** Immunohistochemistry results showing Ki-67 positivity. **f** In situ hybridization results showing EBER positivity. All images are shown at 400 × magnification. Scale bar, 50 μm

Diagnostic aspiration of the right vitreous was subsequently performed. The cytometric bead array results showed that the ratio of interleukin (IL)-10/IL-6 in the vitreous humour was 1.05, slightly exceeding the upper normal limit (usually set as 1). Specifically, the IL-10 level in the vitreous humour was 39.4 pg/mL, while the IL-6 level was 37.5 pg/mL. The vitreous humour was positive for Epstein–Barr virus (EBV) DNA as determined by the polymerase chain reaction (9.90E + 03 copy/mL), and the vitreous was negative for EBV-IgG. Genetic testing by next-generation sequencing of the vitreous humour revealed several oncogenic mutations, including *TP53, JAK2,* and *FAS*. Vitreoretinal lymphoma of the right eye was suspected. Subsequently, whole-body positron emission tomography (PET-CT) showed a hypermetabolic nodule in the retroperitoneal left adrenal area, suggesting a malignant tumour. A core-needle biopsy of the retroperitoneal mass revealed malignant morphological features, including an irregular karyotype, karyokinesis, and karyorrhexis (Fig. 4a). Further immunohistochemistry and fluorescence in situ hybridization (by a Nikon camera and software, × 400 magnification. Scale bar, 50 μm) proved that the lump was ENKTL; the lump was CD7 + , CD2 + , CD3ε + , CD5-, T-cell intracellular antigen 1 (TIA-1) + , Granzyme B (GrB) + , Ki-67 + and EBV-encoded mRNA (EBER) + (Fig. 4b-f). Considering the homology of the two lesions, this patient was confirmed to have metastatic vitreoretinal lymphoma secondary to retroperitoneal ENKTL. Moreover, the positive EBV testing result and oncogenic mutations related to T-cell-derived lymphomas supported the diagnosis. Lymphomatous infiltrates are commonly confined to the space between the RPE and Bruch's membrane in metastatic intraocular ENKTL [6]. Her nasopharyngeal area, a common site for ENKTL, was specifically examined to exclude possible lesions.

Subsequently, the patient received radiotherapy and sintilimab-based chemotherapy treatment at a tumour specialist hospital. Moreover, 0.4 mg of methotrexate was intravitreally injected into her right eye once a week for two months and once a month for the following three months. At the time of submission, the vision of her right eye was improved from 20/125 to 20/32, and the vitreous opacity was significantly improved, as demonstrated in the fundoscopy photos (Fig. 1c, d). OCT at follow-up revealed irregular humps inward into the RPE, while Bruch’s membrane remained intact (indicated by the arrowheads in Fig. 2c, d). PET-CT reexamination showed that the retroperitoneal nodule decreased in size following treatment, and no new lesions were found.

## Discussion and conclusions

VRL, a manifestation of central nervous system lymphomas, occurs in the vitreous and retina and often masquerades as chronic uveitis or vitritis [7]. The majority of VRLs are classified as DLBCL, and most non-B-cell derived intraocular lymphomas are secondary to systemic T-cell lymphoma; among them, primary or metastatic intraocular NK/T-cell derived lymphomas are relatively rare.

ENKTL is an aggressive type of non-Hodgkin's lymphoma (NHL) derived from NK cells or cytotoxic T cells [8, 9]. It is an extremely rare type of lymphoma, accounting for only 0.3% of NHLs in North American and European countries, whereas it occurs more often in Asian, Central, and South American populations [10]. The adjusted ENKTL incidence rate was calculated to be 0.036 per 100,000 individuals (age-adjusted to the US population in 2000) [11]. ENKTL, previously known as “lethal midline granuloma”, typically invades the nasopharynx and upper aerodigestive tract (> 80% of cases) and results in nasal obstruction, epistaxis, and necrotizing lesions of the nose or hard palate [12–14]. ENKTL rarely originates from nonnasal sites, such as the skin, testis, and gastrointestinal tract; these tumours have morphological and immunophenotypical features similar to those of nasal ENKTL [15–17]. EBV-infected lymphoma cells typically characterize all ENKTLs regardless of the site of origin [18].

Limited cases of ocular ENKTL have been reported. The majority of the reported ocular ENKTL cases occurred in the ocular adnexal or obit regions [19–29]. Intraocular involvement, whether primary or metastatic, is rather infrequent. To the best of our knowledge, only three cases of primary vitreoretinal ENKTL have been reported to date [30–32]. Metastatic intraocular tumours commonly develop secondarily to invasion from the nasopharynx [33–35]. Only two cases of metastatic intraocular ENKTL without infiltration of the nasal cavity or paranasal sinuses have been reported previously [6, 36]. In this study, a case of vitreoretinal ENKTL secondary to the retroperitoneum, without nasal lesions, was described. Similar to a published report, metastatic lymphomatous infiltrates were confined to the space between the RPE and Bruch's membrane, which remained intact [6]. Based on the monophyletic diagnostic theory, researchers have proposed that vitreoretinal and retroperitoneal tumours are homologous.

As an invasive and devastating malignancy, ENKTL is characterized by a rapid progression and a dismal prognosis [2]. For patients with relapsed/refractory diseases, the median postprogression survival time is reported to be less than 8 months [37]. Extranodal involvement is predictive of a worse prognosis, and the patient may deteriorate rapidly despite active treatment [38]. Comparatively, patients diagnosed with intraocular DLBCL have better survival outcomes, as a retrospective study of 83 immunocompetent primary intraocular lymphomas (PIOL) patients reported a progression-free survival time of 29.6 months and an overall survival time of 58 months [39]. The diverse outcomes of ENKTL and DLBCL highlight the significance of a definitive diagnosis, which is important for selecting an appropriate treatment plan and making prognostic predictions. At present, the intraocular biopsy is the gold standard for the diagnosis of VRLs [40]; however, the acquisition of qualified specimens by vitrectomy or needle aspiration of subretinal material, or in a minority of cases, full-thickness chorioretinal tissues, is not easy. This invasive procedure is often unacceptable for patients, and early infiltration of the focal point increases the difficulty of the operation. Notably, vitreous biopsy specimens must be transported and handled quickly and gently, as lymphoma cells undergo morphological degradation within 60 min [41]. The use of glucocorticoid treatment can result in a paucity of cells and should be discontinued at least 2 weeks prior to the procedure [42]. False-negative cytology results were reported in approximately 30% of vitreous biopsy specimens collected from a referral centre [43]. In some cases in which biopsies are lacking, as in our case, performing multiple examinations in combination could help discriminate NK/T-cell-derived lymphoma from B-cell-derived lymphoma and lead to a correct diagnosis and an accurate prognostic prediction (Table 1).

**Table 1:** Multiple parameters used to compare the diagnoses of vitreoretinal ENKTL and DLBCL

Parameters	ENKTL	DLBCL
Biopsy	Necrosis, anabrosis, vascular invasion, and polymorphic tumour cells	Diffuse tumorous large B lymphocytes with nuclei approximately twice the size of those of normal lymphocytes
Cytologic examination	CD2 + , surface CD3-, cytoplasmic CD3ε + , CD56 + , TIA-1 + , GrB +	CD20 + , CD19 + , CD22 + , CD79a + , and CD45 +
EBER	100% positive	Approximately 10% positive
IL-10/IL-6	Within the normal limits of ISOLD	Beyond the threshold of ISOLD
Genetic mutations	*JAK3, DDX3, TP53, ASXL1, MLL*, etc	*MYD88, BCL6, IGH* rearrangement
Ophthalmic imaging	Localized spotty hypofluorescence on ICGA or FFA and focal hyperreflection on OCT	

*ENKTL* extranodal natural killer/T-cell lymphoma, nasal type, *DLBCL* diffuse large B-cell lymphoma, *TIA-1* T-cell intracellular antigen 1, *GrB* Granzyme B, *EBER* EBV-encoded mRNA, *ISOLD* Interleukin Score for intraocular Lymphoma Diagnosis, *ICGA* indocyanine green angiography, *FFA* fundus fluorescein angiography, *OCT* optical coherence tomography

Currently, the cytologic examination of aqueous or vitreous humoral samples by aspiration is increasingly acceptable for ophthalmologists [44, 45]. Combined with immunohistochemistry or flow cytometry, the sensitivity and specificity of cytology have become acceptable [46]. Specifically, NK/T-cell-derived neoplastic cells express the typical immunophenotype of CD2 + , surface CD3-, cytoplasmic CD3ε + , CD56 + and cytotoxic (TIA-1, GrB) + molecules [13]. However, B-cell-derived lymphomas typically exhibit pan-B-cell antigen positivity, including CD20, CD19, CD22, CD79a, and CD45 [47]. In this case, immunophenotypic analysis of the retroperitoneal lump revealed a profile of CD2 + , cytoplasmic CD3ε + , CD5-, TIA-1 + , GrB + , and Ki-67 + , which was in accordance with the ENKTL diagnosis. Nevertheless, diagnostic aspiration remains challenging due to the scarcity of lymphoma cells and the excess of normal reactive lymphocytes, along with the fragility of lymphoma cells, which are easily damaged by mechanical trauma [48].

EBV is a double-stranded DNA-containing human herpesvirus, namely, HHV-4 [49]. Typically, EBV affects B cells through CD21 receptors and occasionally affects T cells, NK cells, or monocytes. Remarkably, EBV infection is invariably associated with ENKTL. Currently, detection of EBER by in situ hybridization is required for the diagnosis of ENKTL [50, 51]; this examination was performed in our case, and the results were positive. In lymphoma cells, EBV is homogeneous and clonal, as evidenced by terminal repeat sequences. Thus, it suggests that EBV infection occurs either before or at the same time point as lymphomagenesis [52], implying that EBV infection likely plays an essential role in NK cell lymphomagenesis. While the quantification of circulating EBV DNA is helpful for the assessment of the lymphoma load, it is not specific for diagnosis [53]. In ENKTL patients, EBV DNA fragments < 500 base pairs are released into peripheral blood as the affected neoplastic cells undergo apoptosis. In addition, as B cells are the main reservoirs of EBV in vivo, several B-cell-derived diseases, including DLBCL, Hodgkin lymphoma, and Burkitt lymphoma, could be attributable to the persistent EBV infection of cells. Remarkably, up to 10% of DLBCL tumour tissues are revealed to be EBV + , which indicates that EBER positivity cannot be regarded as an exclusion criterion for DLBCL [54]. Only 1 of 13 specimens from patients with primary intraocular DLBCL were reported to be EBV + , indicating a relatively low positive rate among intraocular DLBCL cases [55]. In summary, EBV-positive VRL should highlight the need to consider a diagnosis of ENKTL; however, we should not reject the possibility of DLBCL.

The assessment of cytokines in the aqueous and vitreous humor regions was recently accepted to improve the diagnostic yield. IL-10 is a B-cell autocrine cytokine that promotes differentiation and function. In malignant B cells, IL-10 has been validated to function by stimulating B-cell antibody production and immune surveillance escape [56, 57]. Elevated IL-10 levels in the vitreous were first proposed as a characteristic for distinguishing PIOL from uveitis in 1995 [58], and this phenomenon was widely validated in subsequent studies [59–61]. Likewise, measurement of IL-10 in the aqueous by AC penetration also indicates the possibility of VRL, but the variation is more subtle [62]. Notably, most of the PIOL cases included were B-cell-derived, and the elevation of IL-10 is not applicable for NK/T-cell lymphomas. Several studies have suggested an optimal threshold of IL-10 for the diagnosis of VRLs; for example, Cassoux et al. established a cut-off value of 400 pg/mL for vitreous IL-10, which yielded a specificity of 0.99 and a sensitivity of 0.8 [62]. In contrast, increasing IL-6 levels in the vitreous is associated with nonmalignant intraocular inflammation. Hence, the IL-10/IL-6 ratio is also proposed as a diagnostic criterion for intraocular DLBCL and typically has an acceptable threshold of 1 [63]. Referring to reported intraocular ENKTL cases, the level of IL-10 is usually not elevated, and the ratio of IL-10/IL-6 is commonly lower than 1, similar to that in cases of uveitis [64]. In our case, the levels of both IL-10 and IL-10/IL-6 in the vitreous were slightly higher than the threshold, which was potentially misleading for the DLBCL diagnosis. However, the newly proposed Interleukin Score for intraocular Lymphoma Diagnosis (ISOLD) system, which adopted a complex formula for detecting B-cell intraocular lymphoma, performs better at distinguishing ENKTL from DLBCL [65]. Therefore, we suggest using ISOLD, rather than a simple ratio or value, to distinguish intraocular DLBCL from ENKTL or uveitis.

In recent years, advances in the technologies and applications of gene sequencing have enhanced the mutational landscape of lymphoma. As reported, *JAK3* mutations, which cause abnormal activation of the JAK/STAT pathways, were verified in 35% of NK/T-cell lymphoma cases [66]. Similarly, *DDX3* mutations were identified in approximately 20% of 105 patients. Other mutations, including *TP53*, *ASXL1*, and *MLL*, were also verified by exome sequencing [67]. In terms of DLBCL, approximately one-third of DLBCL cases carry chromosomal translocations that overexpress *BCL6,* a transcription factor promoting B-cell differentiation and maturation [68]. *MYD88* mutations, especially the recurrent gain-of-function the L256P variant, are a distinguishing feature of activated B-cell diseases such as DLBCL and lead to constitutive NFκB pathway activation [69]. A report revealed 94 *MYD88* mutants among 361 DLBCL cases [69]. *IGH *gene rearrangement is also common in DLBCL [70]. The genetic mutations in the patient reported herein were in accordance with previous findings and included mutations of *JAK2* and *TP53* but not *MYD88* and *BCL6*. In addition, knowledge of the VRL genetics not only improves the diagnostic accuracy but also provides further insights into disease classification, survival prognostication, and therapeutic options [71–74].

Ophthalmic imaging studies, although not specific for diagnosis, usually provide initial clues regarding the VRL. The localized spotty hypofluorescence on ICGA or fundus fluorescein angiography (FFA) and focal hyperreflections on OCT resulting from the subretinal infiltration should catch the attention of the ophthalmologist. As a noninvasive technique, OCT provides high resolution and quantitative measurements of retinal layers, promoting the assessment and diagnosis of intraocular diseases [75]. As reported, the overall positive predictive value of primary intraocular NHL by ocular imaging was 88.9%, and the negative predictive value was 85% [76]. Hence, ocular fundus performance does not help discriminate ENKTL from DLBCL; however, it can help validate the clinical suspicion. Subsequent PET-CT is required to exclude occult nasal ENKTL and scattered lesions [77]. In addition, it is important for accurate staging and prognostic estimation [78].

Generally, intraocular lymphoma has two clinically recognized patterns, vitreoretinal and uveal involvement. Metastatic systemic B-cell lymphoma usually involves the uvea through hematogenous spread via choroidal circulation, while systemic T-cell lymphoma preferentially metastasizes to the vitreous and retina [79]. No conclusion has been reached regarding the route of T-cell lymphoma metastasis. In a similar case of metastatic retinal lymphoma, lesions were localized along retinal arterioles and rested on the RPE, indicating entry via the retinal circulation [80]. The intraocular immune-privileged environment, along with the permissive endothelial receptors, allows for the entry of malignant cells into the retina, with Bruch's membrane serving as a barrier to further spread [81, 82].

An online search of the PubMed database yielded information regarding the case reports and case series of intraocular lymphomas using the MeSH terms intraocular lymphoma, vitreoretinal lymphoma, natural killer T lymphoma and choroidal lymphoma, and metastatic intraocular lymphoma. The natural language terms to identify additional references from preindexed PubMed content and references that were not indexed by these MeSH terms included 'eye or ocular neoplasms' and 'ocular or vitreous tumour'. The retrieved references were indexed to identify additional terms. Embase.com was also searched using the same strategy. This literature search was carried out by two assessors independently.

The majority of VRLs, which often masquerade as chronic uveitis or vitritis, are diagnosed as DLBCL. Although extremely rare, intraocular ENKTL progresses more rapidly and has a worse prognosis than DLBCL and should be treated differently. The biopsy or cytological examination of malignant lesions is vital for a definitive diagnosis but is difficult to perform. Under this condition, multiple parameters in combination, including EBV detection, cytokine assessment, and gene sequencing of intraocular fluid, could help ophthalmologists make the correct diagnosis. More attention and further efforts should be directed at intraocular ENKTL, a rare but fatal disease.

## Data Availability

The datasets supporting the conclusions of this article are available in the National Genomics Data Center repository (HRA002096).

## Abbreviations

VRL: Vitreoretinal lymphoma
DLBCL: Diffuse large B cell lymphoma
NK: Natural killer
ENKTL: Extra-nodal natural killer/T-cell lymphoma, nasal type
OCT: Optical coherence tomography
ICGA: The indocyanine green angiography
AC: Anterior chamber
RPE: Retinal pigment epithelium
IL: Interleukin
EBV: Epstein-Barr virus
PET-CT: The positron emission tomography
TIA-1: T-cell intracellular antigen 1
GrB: Granzyme B
EBER: EBV-encoded mRNA
NHL: Non-Hodgkin's lymphoma
PIOL: Primary intraocular lymphomas
ISOLD: The Interleukin Score for intraocular Lymphoma Diagnosis
